# Human Antibody Responses to the *Anopheles* Salivary gSG6-P1 Peptide: A Novel Tool for Evaluating the Efficacy of ITNs in Malaria Vector Control

**DOI:** 10.1371/journal.pone.0015596

**Published:** 2010-12-14

**Authors:** Papa Makhtar Drame, Anne Poinsignon, Patrick Besnard, Sylvie Cornelie, Jacques Le Mire, Jean-Claude Toto, Vincent Foumane, Maria Adelaide Dos-Santos, Mbacké Sembène, Filomeno Fortes, Francois Simondon, Pierre Carnevale, Franck Remoue

**Affiliations:** 1 UR016 « Contrôle et Caractérisation des Populations de Vecteurs », Institut de Recherche pour le Développement, Cotonou, Benin; 2 UR016 « Contrôle et Caractérisation des Populations de Vecteurs », Institut de Recherche pour le Développement, Montpellier, France; 3 Malaria Control Program-SONAMET Clinic, Lobito, Angola; 4 Laboratoire de Recherche pour le Paludisme, Organisation de Coordination pour la lutte contre les Endémies en Afrique Centrale, Yaoundé, Cameroun; 5 Department of Animal Biology, Cheikh Anta DIOP University, Dakar, Senegal; 6 Malaria Control Program, Ministry of Public Health, Luanda, Angola; 7 UMR145 « VIH/SIDA et maladies associées », Institut de Recherche pour le Développement, Montpellier, France; Université de Toulouse, France

## Abstract

To optimize malaria control, WHO has prioritised the need for new indicators to evaluate the efficacy of malaria vector control strategies. The gSG6-P1 peptide from gSG6 protein of *Anopheles gambiae* salivary glands was previously designed as a specific salivary sequence of malaria vector species. It was shown that the quantification of human antibody (Ab) responses to *Anopheles* salivary proteins in general and especially to the gSG6-P1 peptide was a pertinent biomarker of human exposure to *Anopheles*. The present objective was to validate this indicator in the evaluation of the efficacy of Insecticide Treated Nets (ITNs). A longitudinal evaluation, including parasitological, entomological and immunological assessments, was conducted on children and adults from a malaria-endemic area before and after the introduction of ITNs. Significant decrease of anti-gSG6-P1 IgG response was observed just after the efficient ITNs use. Interestingly, specific IgG Ab level was especially pertinent to evaluate a short-time period of ITNs efficacy and at individual level. However, specific IgG rose back up within four months as correct ITN use waned. IgG responses to one salivary peptide could constitute a reliable biomarker for the evaluation of ITN efficacy, at short- and long-term use, and provide a valuable tool in malaria vector control based on a real measurement of human-vector contact.

## Introduction

Vector-borne diseases constitute major public health problems in developing countries and currently present high risks of re-emergence in the developed world. Developing tools for disease control is a priority, especially new anti-vector strategies to prevent transmission. Among these diseases, malaria represents the greatest worldwide problem, causing at least 400 million acute cases every year with around 1 million deaths [1]. Most of these deaths occur in children from Sub-Saharan Africa and are due to *Plasmodium falciparum* species. In these areas, the *Anopheles gambiae* complex is the major vector [2].

Preventive methods are used against both parasite (chemoprophylaxis) and vector (insecticide-based control). Among anti-vector strategies, Insecticide Treated Nets (ITNs) are currently the most efficient strategy for reducing human exposure to the vector, *Plasmodium* transmission and malaria morbidity [3], [4], [5]. When correctly used, even moderate coverage of populations (35–65%) can afford substantial community benefit as well as personal protection [6], [7], [8], [9]. Moreover, implementation of Long-Lasting Insecticidal Nets (LLINs) represents an achievable means of rapidly improving ITN coverage [10]. The evaluation of ITN efficacy is currently based on entomological methods (entomological inoculation rate, *Anopheles* abundance and agressivity) and, in humans, on parasitological and clinical assessments [4], [11], [12]. The reference WHO method for phase 3 evaluation of ITNs efficacy is based on the measurement of *P. falciparum* density in human populations [12]. However, these methods present limitations when it comes to large-scale field studies, especially when transmission rates and exposure levels are low (dry season, high altitude, urban settings or after vector control). Moreover, evaluating *Plasmodium* density in human individuals is labour-intensive by active follow-up of populations. Entomological methods are mainly applicable at the population/area level and do not give a measure of the heterogeneity of individual exposure in a given area. Human-landing catch measurements (adult volunteers) are currently the reference method for evaluating individual human exposure but it raises ethical questions and it may not be relevant to children [13]. In addition, as exposure levels drop with ITNs use, all these monitoring methods become less effective for evaluation by National Malaria Control Programs [14].

In order to improve vector control, much effort is being devoted to developing new indicators to evaluate, at the individual level, the efficacy of control strategies. One promising approach is based on the idea that exposure to arthropod vector bites can be assessed by directly measuring real human-vector contact. Indeed, the human antibody (Ab) response to arthropod salivary proteins could give a measure of exposure to vector bites [15], [16]. At the time of biting, the female mosquito injects saliva containing bioactive molecules which facilitate the blood meal and some of these are antigenic [17], [18], [19]. Human Ab responses to the saliva of various vectors, e.g. *Triatoma* (Chagas' disease) [20], *Ixodes* ticks (Borrelia) [21], [22], phlebotomes (Leishmania) [23], [24] and *Glossina* (African trypanosomiasis) [25] have been reported as reliable immunological markers for vector exposure. For mosquitoes, anti-saliva Ab responses has been related to exposure to *Culex*, *Aedes* [26], [27], [28], [29], *An. gambiae* [30], *An. dirus* [31] and *An. darlingi* [32]. Recently, it has been shown that the Ab response to whole *An. gambiae* saliva could be a useful biomarker for evaluating ITN efficacy in phase 3 studies [33]. Even if this concept may appear to be valid, whole vector saliva could not be used, as pertinent indicator, because of i) potential cross-reactivity with salivary epitopes of other hematophagous arthropods; ii) lack of reproducibility between saliva batches and iii) the adequate production needed for large-scale studies. For use as a biomarker for *Anopheles* exposure, the specific [34], [35] and antigenic [36] *Anopheles* SG6 salivary protein has been identified as an encouraging candidate [37]. The gSG6 protein, first identified in *An. gambiae* [38], was further reported as being highly conserved among *Anopheles* species [39], [40]. To optimize this biomarker candidate, peptide design has recently been applied using bioinformatics approach to generate five *Anopheles* specific peptides (gSG6-P1 to gSG6-P5). Among them, only the gSG6-P1 peptide was validated as a specific biomarker of exposure to malaria vectors. Indeed, the level of human IgG to gSG6-P1 peptide evaluated the level of exposure to *An. gambiae* bites in human populations from a rural area in Senegal [37]. IgG response to this peptide has been also confirmed as biomarker for evaluating very low-level exposure to *An. gambiae* [41] as well as *An. funestus* (the second major malaria vector in Africa) [42]. In addition, the gSG6-P1 peptide can be easily synthesized in large quantity and offers an efficient solution to the lack of reproducibility observed with whole salivary extracts [37].

The present study addresses a potentially important application of such biomarker as a tool to evaluate the efficacy of ITN-based strategies. Human IgG responses to the gSG6-P1 peptide were evaluated before and after the introduction of ITNs in individuals living in a malaria-endemic area. The results focused on the biomarker's potential for evaluating short-term ITN efficacy.

## Materials and Methods

### Ethics Statement

This study was conducted in accordance with the Edinburgh revision of the Helsinki Declaration, and was approved by the National Malaria Control Program of the Ministry of Health of Angola (October 17th 2008), the only one Ethical authority in 2008 for approving studies on malaria research in Angola. Written informed consent (signed by the head of each household) was obtained for all individuals enrolled in the study, by the SONAMET Malaria Control Program (MCP) which supervise/control malaria infection of all workers for SONAMET and their family. This consent procedure was regularly approved by SONAMET workers, beneficing from several malaria studies/survey by MCP, and was approved by the involved Ethical authority in Angola.

### Study population

This study was conducted in Lobito, a coastal city of Western Angola, from March 2005 to January 2007. The site is in the tropical savannah with a rainy season from October to May, with approximately 600–700 millimetres of rain per year. The duration of the malaria transmission season varies between 7 and 12 months with a peak between March and May. The major malaria vector is here the *An. gambiae s.l.* complex [43], [44].

The population has been previously described [33]. Briefly, all workers of the SONAMET company lived in 250 households. Residents of these households were followed in the SONAMET in-patient clinic. In 2004, the presence of malaria parasite was diagnosed in sixty (60) households (positive, at least, in one member of household) by SONAMET Malaria Control Program (MCP). Twenty-one (21) of these 60 households were then randomly selected for the present study. These families lived in the Bella Vista district where exposure to *An. gambiae s.l* bites was relatively uniform. All residents of the 21 selected households, corresponding to two hundred and fifty (250) individuals (children and adults), were included for longitudinal follow-up, with evaluations every 6 weeks from March 2005 to January 2006 (before ITNs), and from April 2006 to January 2007 (after ITNs). The families were given LLIN treated with deltamethrin (Permanet®) in February 2006 (according to the number of rooms and beds per household). At each visit, samples were collected from each individual: thick blood smears for parasitological measurements and dried blood spots (on filter paper) for immunological analysis. Parasite density (parasitaemia) was calculated as the number of *P. falciparum* parasites per microliter of blood; mean parasitaemia values (x+1) were calculated [33]. Immunological tests were performed in a sub-sample (n = 105, children and adults) of the whole study population (n = 230). Individuals who missed more than 2 of the 14 visits (travel, illness, change of household,…) were excluded from the immunological assessments as described previously [33]. Filter papers were kept at +4°C in Silicagel before testing.

### Entomological analysis and survey of ITN use

Mosquitoes were collected every six weeks during the study period at 6 reference households, representative of the studied area. *An. gambiae* density was evaluated using capture by CDC miniature light traps, deployed from 19:00 hours to 07:00 hours for two consecutive nights. PCR was used to confirm species to yield an estimate of the number of *An. gambiae/*trap/night.

After the introduction of ITNs, their use by individuals and their quality were inspected, the night before each blood sampling, by the MCP team. Information were then collected for all studied individuals by questionnaires, covering: i) the number of installed ITNs, ii) the number of exchanged ITNs and iii) the number of damaged ITNs (hole, torn, etc.), as previously described [33].

### Salivary gSG6-P1 peptide

The gSG6-P1 peptide was designed as previously described [37], synthesized and purified (>95%) by Genepep SA (St-Clément de Rivière, France). All peptide batches were shipped in lyophilized form and then suspended in 0.22 µm ultra-filtered water and frozen at −80°C until their use.

### Evaluation of human IgG antibody levels (ELISA)

Standardized dried blood spots (1 cm diameter) were eluted by incubation in 300 µl of phosphate buffered saline (PBS–Tween-0.1%) at 4°C for 24 hours [33]. ELISAs were carried out on eluates to assay IgG to the gSG6-P1 antigen. Maxisorp plates (Nunc, Roskilde, Denmark) were coated with gSG6-P1 (20 µg/mL) in PBS. Each eluate was then incubated (duplicate) at 4°C overnight at a 1/20 dilution (PBS–Tween-1%). This optimal dilution had been determined in preliminary experiments. Mouse biotinylated Ab against human IgG (BD Pharmingen, San Diego, CA, USA) was incubated at a 1/2000 dilution and peroxidase-conjugated streptavidin (Amersham, Les Ulis, France) was then added (1/2000) Colorimetric development was carried out using ABTS (2,2'-azino-bis (3-ethylbenzthiazoline 6-sulfonic acid) diammonium; Sigma, St Louis, MO) in 50 mM citrate buffer (pH 4) containing 0.003% H_2_O_2_. Optical Density (OD) was measured at 405 nm. In parallel, each test sample was assessed in a blank well containing no gSG6-P1 antigen (ODn) to measure non-specific reactions. Individual results were expressed as the ΔOD value: ΔOD = ODx­ODn, where ODx represents the mean of individual OD in both antigen wells. Specific anti-gSG6-P1 IgG levels were also assayed in non-*Anopheles* exposed individuals (n = 14 – neg; North of France) in order to quantify the non-specific background Ab level and to calculate the specific immune response threshold (TR): TR =  mean (ΔDO_neg_)+3SD = 0.204, i.e. an exposed individual was classified as a responder if its ΔOD>0.204.

### Statistical analysis

All data were analyzed with GraphPad Prism4 software® (San Diego,CA, USA). After checking the non-Gaussian distribution, the non-parametric Mann-Whitney U-test was used to compare Ab levels between two independent groups, the Wilcoxon matched pairs test was used for comparison between two paired groups, the non-parametric Kruskal-Wallis test for comparisons between more than two groups and the Chi-2 test to compare two proportions. All differences were considered significant at P<0.05.

## Results

### Anti-gSG6-P1 IgG responses before and after ITN use

**Figure 1:**
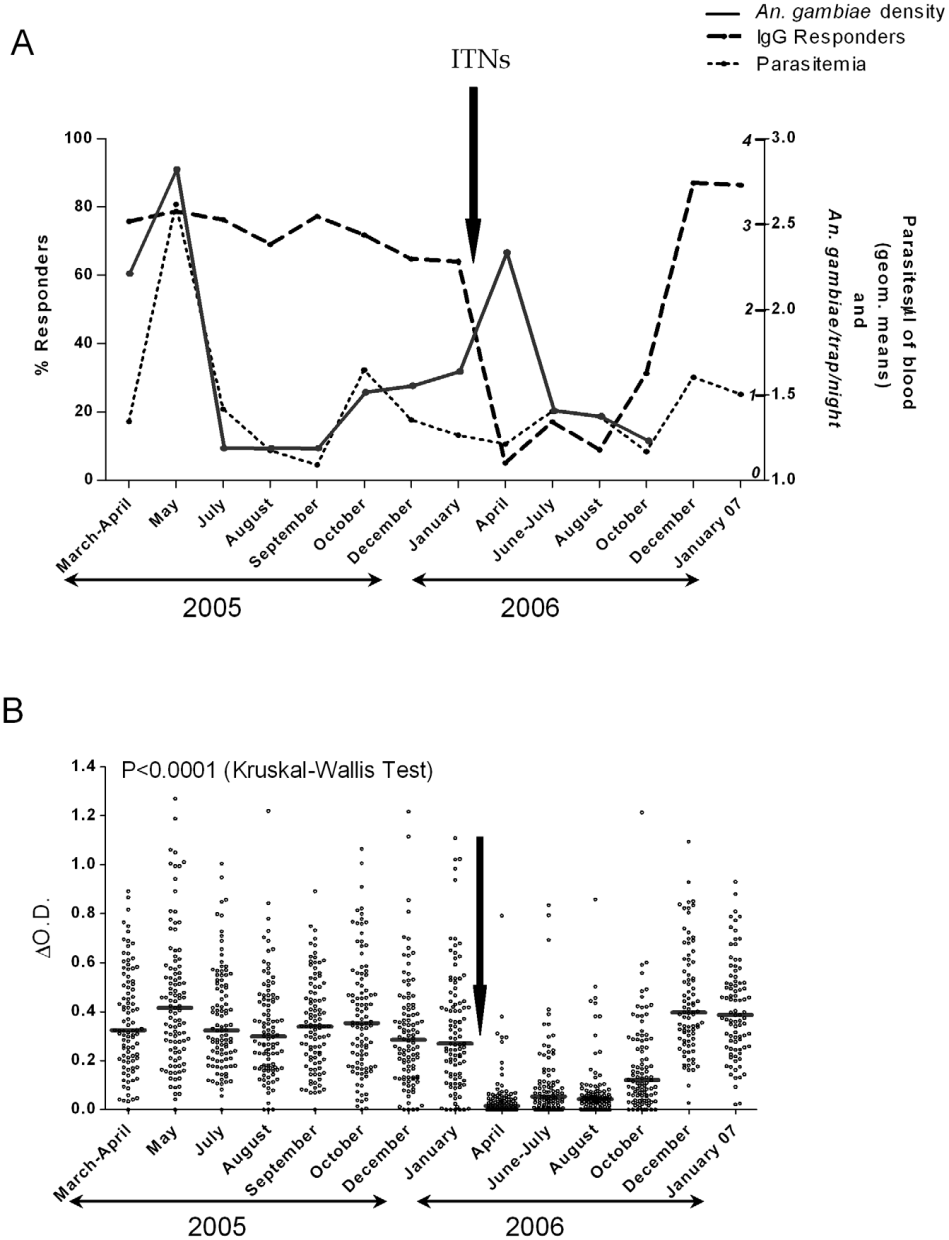
IgG Ab responses to gSG6-P1 before and after ITN use. The percentage (%) of anti-gSG6-P1 IgG immune responders (thick-dotted line) in the “immunological” sub-population (n = 105), before (2005) and after (2006 and January 2007) the installation of ITNs (A). These results are presented together with the intensity of *P. falciparum* infection (mean parasitaemia – fine-dotted line) measured in the same population and the mean of number of *An. gambiae* (solid line) in the studied area (A). Entomological data (number of *An. gambiae*) were not available in December 2006 and January 2007 (the two last months of the study). This arrow indicates the installation of Insecticide Treated Nets (ITNs) in February 2006. Individual anti-gSG6-P1 IgG levels (ΔOD) are presented before (2005) and after (2006) the installation of ITNs (B). Bars indicate the median value for each studied month. Statistically significant differences between months are indicated.

The evolution of the percentage of “immune responders” (Fig. 1A) and the levels of anti-gSG6-P1 IgG Abs (Fig. 1B) were evaluated before (March 2005 to January 2006) and after (April 2006 to January 2007) the installation of ITNs (February 2006).

Before the introduction of ITNs, the percentage of anti-gSG6-P1 IgG responders appeared relatively constant according to months (64 to 81%) (Fig. 1A). Within two months of the ITNs introduction (April. 2006), the percentage of immune responders had decreased significantly (P<0.0001) down to 5% from 64% in January 2006. In parallel, the density of *An. gambiae* (classical entomological methods) and the intensity of *P. falciparum* infection (mean parasitaemia) are shown for the same period (Fig. 1A). Whereas peak of parasitaemia was observed in May 2005, only low-level infection was observed after the introduction of ITNs (2006). In particular, parasitaemia was lower in April 2006 compared to May 2005, which suggests that ITNs were effective, as previously described [33]. In addition, the densities of *An. gambiae* peaked in May 2005 with a small rise from October 2005. After the introduction of ITNs, a similar peak was observed (April to July 2006), indicating that entomological exposure was comparable in 2006 to that in 2005 in the studied area.

A similar trend was observed in the percentage of immune responders (Fig. 1A) and specific IgG Ab levels (Fig. 1B). In spite of inter-individual heterogeneity, the anti-gSG6-P1 IgG level was strong and relatively stable before the introduction of ITNs (Fig. 1B) although small but significant seasonal variations were seen in median specific IgG levels (P<0.0001). The major peak of Ab level was detected in May 2005, as observed for both entomological *An. gambiae* exposure and *P. falciparum* density (Fig. 1A). Immediately after the introduction of ITNs, a marked decrease in anti-gSG6-P1 IgG was observed between April and October 2006. Specific IgG levels were markedly lower in 2006 than in 2005 in June-July (P<0.001), August (P<0.001) and October (P<0.001). Interestingly, the greatest drop in Ab levels was observed in all individuals just two months after ITN introduction in April 2006, the month corresponding to the peak anti-IgG level before ITNs (P<0.001 for April 2006 compared to March-April 2005 and P<0.0001 for April 2006 compared to May 2005). The still positive Ab responses to gSG6-P1 (Fig. 1B) just after ITN installation (April and June-July 2006) were almost exclusively observed in older children and adults: the 5 responders in April 2006 and 15/16 responders in June-July 2006 were from 7-14 and >14 years age groups.

The percentage of specific immune responders (Fig. 1A) and the level of anti-gSG6-P1 IgG Ab (Fig. 1B) increased significantly in October 2006 compared to August 2006 (P<0.001) to reach a high level in December 2006 and January 2007, similar to that observed before ITN introduction in 2005. Interestingly, the major results of the ITN survey (loss rate and correct/damaged-usage rate) evaluated by two horizontal surveys (April and June/July 2006) indicated that: i) after just 4 months (June/July), only 63% of the ITNs were installed, and ii) only 53% were being used correctly (in use and undamaged), as previously described in detail [33].

### Individual IgG responses to gSG6-P1 and short-term ITN efficacy

**Figure 2:**
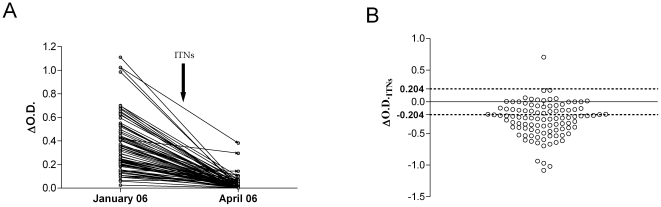
IgG response to gSG6-P1 as biomarker for short-term ITN efficacy. Changes in individual IgG levels (ΔOD) are presented between “just before” (January 2006) and “just after” (April 2006) ITN introduction (n = 105; children and adults) (A). The arrow indicates the installation of Insecticide Treated Nets (ITNs) in February 2006. Individual IgG level changes from January (before) to April are presented (B) by individual ΔOD_ITNs,_ value (ΔOD_ITNs = _ΔOD_April06_, - ΔOD_January06_). The threshold of specific IgG responders (TR = 0.204) is indicated (dotted line). Significant positive (ΔOD>0.204) or negative (ΔOD<−0.204) changes are therefore individually presented.

Previous analyses have been performed at the population level. To investigate changes in anti-gSG6-P1 IgG levels as a biomarker at the individual level and immediately after ITN installation, IgG level was individually evaluated just before (January 2006) and just after (April 2006) the introduction of ITNs (Figure 2). For almost all individuals, specific IgG levels decreased significantly in April 2006 compared to January 2006 (P<0.0001 – Fig. 2A). Other patterns - an increase or no change - were observed in only 5% of individuals. In addition, a new indicator of the January/April difference in IgG level was defined (ΔOD_ITNs_ = ΔOD_April_ ­ ΔOD_January_) to assess individual trends (positive, negative or unchanged) between both months (Fig. 2B). After applying a threshold for positive IgG response (ΔOD>0.204), 60% of individuals showed a decrease (ΔOD_ITNs_ <­0.204) whereas only 1% showed an increase (ΔOD_ITNs_ >0.204) and 39% (39/100) showed no significant change (­0.204<ΔOD_ITNs_ <+0.204).

### Changes in anti-gSG6-P1 IgG responses according to age

**Figure 3:**
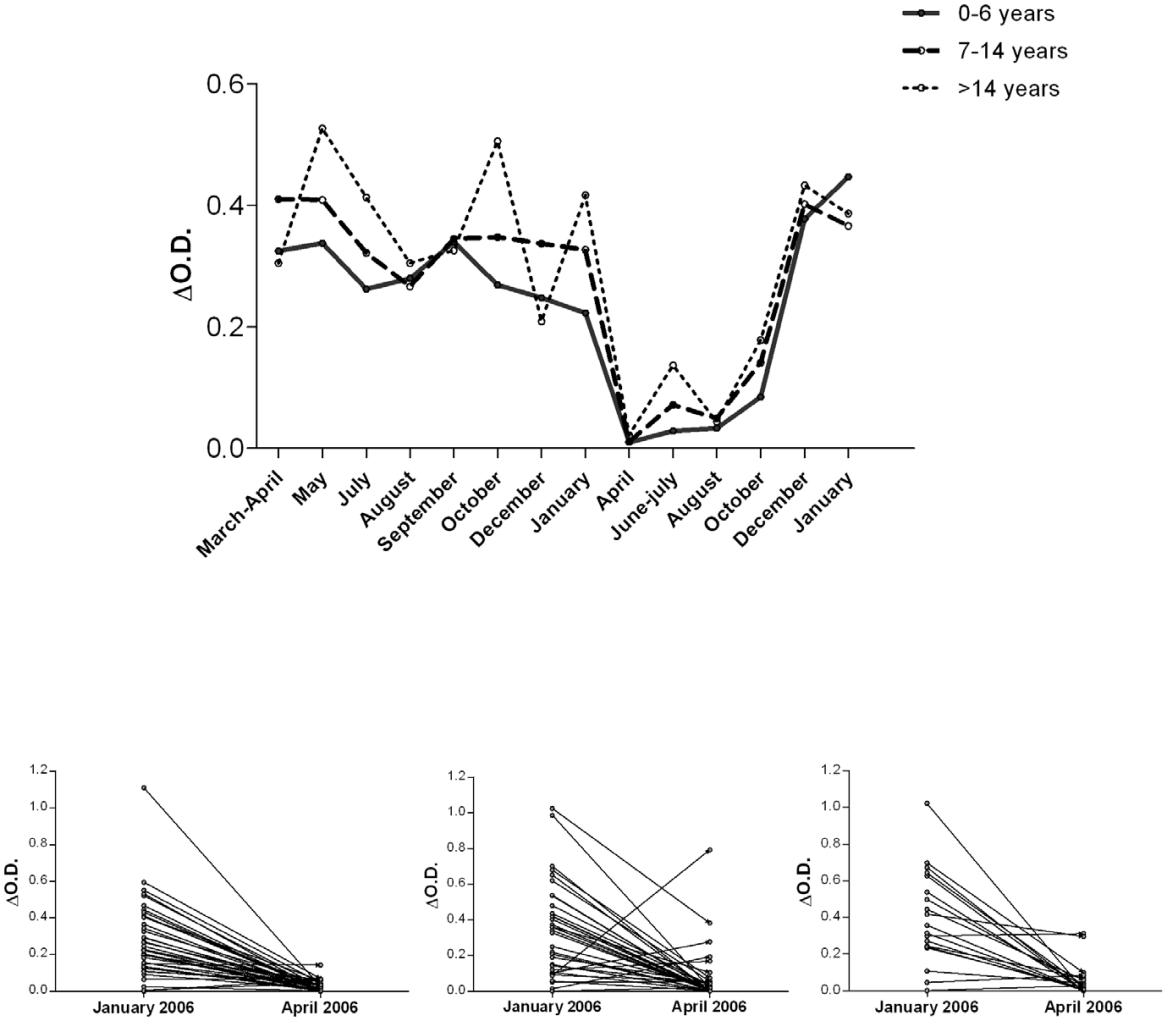
Changes in anti-gSG6-P1 IgG levels before and after the introduction of ITNs according to age group. Median anti-gSG6-P1 IgG levels in 2005–2006 are presented according to three age groups (A): 0–6 years-old (solid line; n = 49), 7–14 years-old (thick-dotted line; n = 34) and >14 years-old (fine-dotted line; n = 25). Individual, short-term changes in specific IgG levels from January (just before) to April (just after ITN installation), are presented according to age group (0–6 years = B; 7–14 years = C and >14 years = D). The arrow indicates the installation of Insecticide Treated Nets (ITNs) in February 2006. Statistically significant differences between age groups are indicated for respective months (*: P<0.05; ***: P<0.0001).

The changes in anti-gSG6-P1 IgG level was analysed (Figure 3) according to age of individuals in 3 age groups: 0–6 (n = 49), 7–14 (n = 34) and >14 years (n = 25). Similar trends in all age groups were observed between 2005 and 2006 (Fig. 3A), in particular a substantial drop in April 2006 after the introduction of ITNs. Nevertheless, there are significant age-related differences in some months, e.g. during the peaks before ITN introduction (May and October 2005) and in June-July 2006, where anti-gSG6-P1 IgG levels were significantly higher in older-children and adults (>14 years) than in younger children. Before ITN installation (2005), seasonal variations were most marked in the >14 years age group.

The relationship between short-term ITN efficacy and anti-gSG6-P1 IgG levels was analysed according to age group (Figure 3 B, C and D). A marked decrease was observed between January and April 2006 in all age groups. In particular, IgG level decreased clearly in all young children from 0–6 years (Fig. 3B) whereas some individuals of 7–14 years (Fig. 3C) and >14 years (Fig. 3D) presented increased or unchanged Ab responses.

## Discussion

This study focused on the application of a new biomarker based on evaluation of the human Ab response to an *Anopheles* salivary peptide, as an indicator for evaluating the efficacy of malaria vector control. In this study, a step-by-step approach was adopted to validate the anti-gSG6-P1 IgG response as a biomarker for ITN efficacy from the whole population level to the individual level with a view to identifying a potential tool for evaluation immediately after the introduction of ITNs.

Firstly, the present study and previous data [33] indicated that the correct use of deltamethrin ITNs was followed by a considerable decrease of *P. falciparum* parasitaemia, the current WHO criterion for vector control efficacy [13]. It indicated that ITNs installation was short-term effective in the studied population. In the whole population, the percentage of immune responders and the level of anti-gSG6-P1 IgG Ab in most individuals decreased considerably just after ITNs introduction (April-October 2006). The drop was particularly marked in April-August 2006, corresponding to the peak of *An. gambiae* exposure. Interestingly, the entomological data indicated that this season-dependent peak was similar before (2005) and after (2006) ITN use. This suggests that ITN installation had no impact on *An. gambiae* density, probably because of the low percentage of the overall population covered in the studied area [45]. It indicated also that the drop of anti-gSG6-P1 IgG response was associated with correct ITN use and not due to low *Anopheles* density. As described [37], [41], Ab responses to gSG6-P1-peptide could accurately reflect human-vector contact. A marked drop after ITN installation was observed in all age groups studied (<7 years, 7–14 years, and >14 years) suggesting that this biomarker is relevant for ITN evaluation in all age groups. Taken together, these results confirm that - as previously shown with whole *An. gambiae* saliva [33] - the estimation of human Ab responses to *Anopheles* salivary proteins could provide a reliable biomarker for evaluating the efficacy of malaria vector control. It validates the general approach to use Ab to salivary antigens for evaluating the quantitative human exposure to mosquito bites.

The specific gSG6-P1 peptide is more reliable than whole saliva which may show cross-reactivity with salivary epitopes from other arthropods and which could skew and/or overestimated described effects [37]. Interestingly, compared to IgG responses to whole saliva [33], the percentage of responders and the levels of anti-gSG6P1 IgG showed less variability before ITN installation (2005) and especially remained high in January 2006, one month before ITN installation. The use of gSG6-P1 peptide increases the sensitivity and the specificity of such type of biomarker. It strengthens such effective peptide biomarker for the evaluation of ITN efficacy at a large scale. The smallness of seasonal variations could preclude the need for demanding longitudinal evaluation as needed for parasitaemia indicator (full year before introduction and especially during the peak of transmission). One measurement of anti-gSG6-P1 Ab just before ITN installation could provide a representative picture of the specific Ab response, i. e. exposure level to *Anopheles*, prior to ITN introduction.

The gSG6 peptides were designed on the basis of the *An. gambiae s.s.* sequence, the only *Anopheles* genome completely available [46]. In the studied area, the *An. gambiae s.l.* complex, especially *An. gambiae s. s.*, is the main vector of *P. falciparum* as previously described [44]. However other *Anopheles* species, such as *An. arabiensis* and *An. melas*, were locally caught in the study area and had been reported to participate to malaria transmission as secondary vectors [44]. The exposure of individuals to these “secondary” *Anopheles* species have been probably evaluated using gSG6-P1 peptide which presents a very stable domain of the gSG6 *Anopheles* salivary protein [37] and monoacids sequences highly conserved among *Anopheles* species [40]. For example, it has been shown that gSG6-P1 peptide shares 82% and 91% identity with *An. stephensi* and *An. funestus* [37] and that IgG response to this peptide was also biomarker of *An. funestus* exposure [42]. All these data tends to support the idea that gSG6-P1 can be used to evaluate the exposure to major *Anopheles* species known to be vector of malaria.

After an analysis at the whole population level, an ideal biomarker has to be used i) at the individual level and ii) to evaluate short-term ITN efficacy. The individual results showed that few individuals presented a positive anti-gSG6-P1 IgG response just after ITN installation (from April to August 2006) and that most of these were older children or adults (>14 years). It cannot be ruled out that these older individuals may stay outside the house until late in the evening when *Anopheles* starts biting again so they therefore derive less protection from ITNs than young children who go to sleep earlier in the evening. Individual specific IgG response between January (before) and April 2006 (2 months after ITNs) showed a considerable decrease in all age groups. This rapid decrease after correct ITN usage appears to be a special property of the anti-saliva Ab response which is short-lived in the absence of ongoing antigenic stimulation, at all ages [30], [36], [47]. The response does not seem to build up but wanes rapidly, when exposure failed. This property represents a major strength for use such salivary biomarker for evaluating the human exposure to mosquito bites, especially for efficacy of vector control. In addition, using a response threshold (ΔOD = 0.204) combined with ΔOD_ITNs_ - the difference between April (after ITNs) and January 2006 (before) - makes possible the use of this operational biomarker large-scale [40]. If the ΔOD_ITNs_ value is between ­0.204 and +0.204, no clear difference can be defined. In contrast, if the individual ΔOD_ITNs_ value <−0.204, it could be concluded with a high level of confidence that this individual is benefiting from ITN installation. The ΔOD_ITNs_ parameter could therefore provide a measure of ITN efficacy at the individual level. An individual biomarker would also be relevant at the large-scale, operational level in the field, e.g. in National Malaria Control Programs (NMCP), since it is pertinent for the evaluation of short-term ITN efficacy, as demonstrated in the study (April, 2 months after ITN installation). Short-term and individual changes in Ab responses to gSG6-P1 provide a superior biomarker to *P. falciparum* parasitaemia, especially in a context of low malaria transmission and by evaluation of ITN efficacy, only in small sub-sample (around N = 100) from populations receiving ITN (parasitaemia needing a larger population of the order of N = 1000). We have previously shown the high sensitivity and specificity of the gSG6-P1 Ab response make it ideal for the evaluation of low-level exposure to *An. gambiae* [41] which is relevant in areas where exposure/transmission is being curtailed by NMCP efforts. In addition, its evaluation can be performed from blood spots on filter paper, which is easy and operational to be integrated in NMCP strategies.

The ITN survey, as previously reported in the studied area [33], indicated that only 53% of ITNs were being correctly used and were undamaged after just 4–5 months of use (June/July 2006), as also observed in several other endemic areas [14]. Because of the high sensitivity of gSG6-P1, we were able to observe an increase in specific IgG levels from October 2006 (8 months after ITN installation). The strong decrease of anti-gSG6-P1 IgG response was therefore transient after ITN installation and the biomarker reappeared as correct ITN usage waned and individuals were once re-exposed to *An. gambiae*. These results point up the relevance of this salivary biomarker for the evaluation of short-term efficacy as well as longer-term monitoring.

The results suggest that this salivary biomarker for *Anopheles* exposure could constitute an efficient, reliable and new tool for evaluating the efficacy of malaria vector control, at both population and individual levels. Further studies are however needed to confirm this in other areas and for different vector control strategies. Finally, this first approach could be similarly applied to vector-control strategies for other mosquito-borne diseases such as emergent arbovirus diseases.
